# Predicting seismic-induced liquefaction through ensemble learning frameworks

**DOI:** 10.1038/s41598-019-48044-0

**Published:** 2019-08-13

**Authors:** Mohammad H. Alobaidi, Mohamed A. Meguid, Fateh Chebana

**Affiliations:** 1 Civil Engineering and Applied Mechanics, McGill University, 817 Sherbrooke Street West, Montréal, QC H3A 0C3 Canada; 2 Eau Terre Environnement, Institut National de la Recherche Scientifique, 490 Rue de la Couronne, Québec, QC G1K 9A9 Canada

**Keywords:** Natural hazards, Structural geology

## Abstract

The regional nature of liquefaction records and limited information available for a certain set of explanatories motivate the development of complex prediction techniques. Indirect methods are commonly applied to incidentally derive a hyperplane to this binary classification problem. Machine learning approaches offer evolutionary prediction models which can be used as direct prediction methods to liquefaction occurrence. Ensemble learning is a recent advancement in this field. According to a predefined ensemble architecture, a number of learners are trained and their inferences are integrated to produce stable and improved generalization ability. However, there is a need to consider several aspects of the ensemble learning frameworks when exploiting them for a particular application; a comprehensive evaluation of an ensemble learner’s generalization ability is required but usually overlooked. Also, the literature falls short on work utilizing ensemble learning in liquefaction prediction. To this extent, this work examines useful ensemble learning approaches for seismic-induced liquefaction prediction. A comprehensive analysis of fifteen ensemble models is performed. The results show improved prediction performance and diminishing uncertainty of ensembles, compared with single machine learning models.

## Introduction

Seismic-induced liquefaction of soils is one of the major ground failure consequences of earthquakes. In general, liquefaction is the transformation of soil from a solid to a liquefied state as a result of increased pore water pressure, which commonly occurs during sudden and massive shaking of the ground. This phenomenon leads to catastrophic loss of lives and irreversible damage to critical infrastructure. Predicting liquefaction susceptibility is, hence, considered a major research frontier in geotechnical earthquake engineering^1,2^.

Commonly used approaches in liquefaction prediction are sometimes classified into two broad clusters, deterministic (or semi-empirical) approaches and empirical approaches^3^. In deterministic studies, the researchers report various degrees of experimental and *in-situ* testing setups, where a characterization of susceptibility to liquefaction is concluded. Empirical approaches, on the other hand, aim to quantify the potential of liquefaction using raw variables obtained from different sites across the globe. These methods put more emphasis on answering the question of liquefaction/no-liquefaction rather than relating the variables of interest to each other analytically. Furthermore, we observe that the literature provides little distinction between the two clusters and that development in each cluster is carried out unilaterally^4–6^. Ideally, deterministic techniques can be used to support empirical models through identifying appropriate features to liquefaction; however, little cross-examination of these approaches has been reported to date^7,8^.

**Figure 1:**
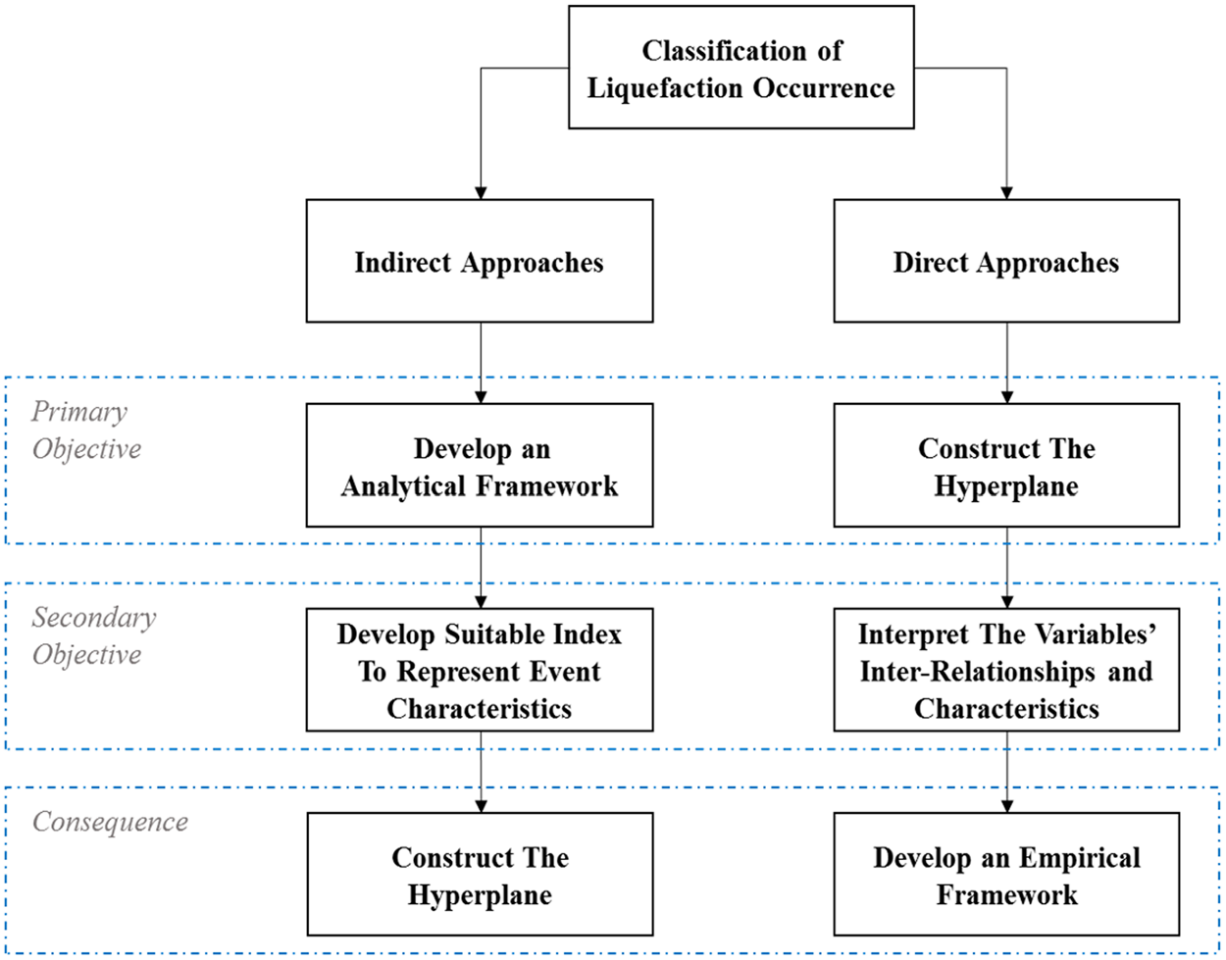
A summary of the general classification approach utilized in the two types of research methodologies for evaluating the liquefaction potential.

While this work targets the development of robust and more stable classification models using the available data and explanatory variables, we attempt to make a distinction between the two broad approaches to studying liquefaction susceptibility. We use the terms direct and indirect modeling approaches to refer to empirical and deterministic studies, respectively. The motivation behind this nomenclature is related to the classification objective (Fig. 1). Direct models attempt to explicitly establish a separating hyperplane, also referred to as the decision boundary or the limit-state. On the other hand, indirect models derive useful transformations which deduce important relationships between variables of interest; a liquefaction-triggering mechanism can then be indirectly inferred from the variables of interest. Indirect models incidentally formulate the classification problem with the benefit of providing conceptual interpretation of the derived index^9,10^. Remarkably, such approaches can be considered as unsupervised learning techniques from a machine learning perspective, where the liquefaction identity of the considered case study does not take part in the development of the classification approach.

In studies utilizing direct approaches, a supervised learning scheme is developed, where explanatory variables and predetermined knowledge of the liquefaction events are exploited in the construction of the hyperplane. Statistical models are commonly used in producing direct inferences about the likelihood of liquefaction, given some soil-related as well as earthquake-related information. Logistic Regression, Probabilistic Regression and Naïve Bayes Filters are examples of such models^3,11,12^. Nevertheless, more advanced prediction methods are required to provide better generalization ability over a wide range of liquefaction observations, rather than local thresholding of the phenomenon through filtering already limited datasets.

Recently, supervised machine learning techniques have been proposed in the literature and provided superior performance in learning complex relationships while maintaining a reliable generalization ability. The most notable machine learning models used in seismic-induced liquefaction studies are Support Vector Machines (SVMs)^13^, Decision Trees (DTs)^14^, Artificial Neural Networks (ANNs)^1^ and Extreme Learning Machines (ELMs)^15^.

Supervised learning is of empirical nature and requires available information to create functional relationships between the explanatories and the target variable. Several drawbacks are usually identified from utilizing machine learning techniques, such as overfitting and unstable performance. However, the availability of computational resource nowadays further motivates the creation of more complex techniques which provide far better generalization ability than their predecessors^16,17^.

**Figure 2:**
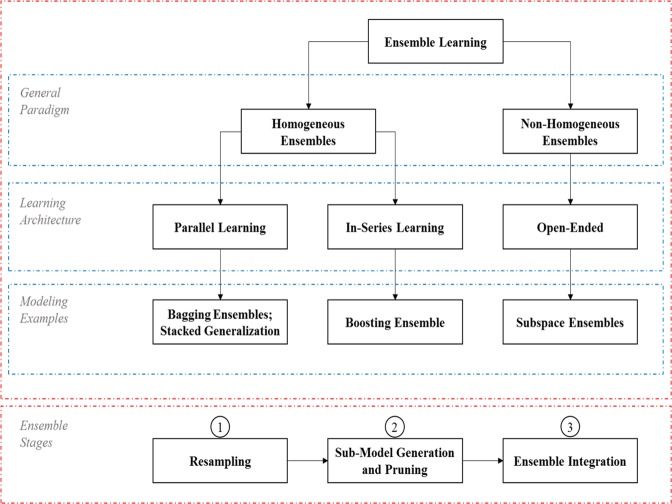
The main divisions (top red rectangle) and learning stages (bottom red rectangle) of ensemble learning.

Ensemble learning, a recent advancement in machine learning, is defined as the process of generating multiple prediction models which are trained using subsets of the available data and then fused to make a prediction. Ensemble learning not only produces a more stable global model, but also guarantees diminishing uncertainty^18^. Continuous work has been published in the broad literature, discussing the effectiveness of ensemble learning^19–21^. Generally, for a learning framework to be considered an ensemble model, it should have three fundamental stages (Fig. 2). The first stage is resampling^22^, which consists of generating a number of subsets of data resamples from the original sample set. The second stage is sub-ensemble model generation and pruning, which is concerned with choosing appropriate individual models for the system of interest. The third stage is ensemble integration, which merges estimates produced by the sub-ensemble models to determine the ensemble estimate. Ensemble learning frameworks are divided into two broad clusters, homogeneous ensembles and non-homogeneous ensembles^23–25^. In homogeneous ensemble frameworks, ensembles adopt the same resampling technique, the same version of a certain model, and only one integration technique^26,27^. Non-homogeneous ensemble frameworks violate the definition of homogenous ensembles, but maintain the three fundamental stages of ensemble learning^25,27^. In this work, homogeneous ensembles are considered and, as a result, all the individual models will be of the same type and input/output configuration.

One of the major research frontiers in ensemble learning is the modeling of ensemble-based diversity, which is theorized to create stable and enhanced generalization ability of ensembles over individual models^28–30^. Diversity in learning is defined as the amount of variation existing between the resulting sub-ensemble models^31,32^. The nature of the resamples is normally described as a first source of ensemble diversity^23,33^, which typically manifests in the training stage. The individual models and the ensemble integration stage are considered secondary sources of diversity^34,35^. Recent investigation of this concept has led to important breakthroughs in the development of quantum ensemble learning^36^.

In liquefaction prediction studies, limited adherence to proper utilization of machine learning has been observed, not to mention the deficiency in reporting the uncertainty and performance stability of the used models over the considered case studies. Also, little attention has been paid to the recent development in supervised learning techniques. This work presents different ensemble learning frameworks and examines their capability in liquefaction prediction. In addition to Logistic Regression, four different machine learning techniques, namely SVM, DT, ELM, and ANN, are used as sub-ensemble models. Three different ensemble learning frameworks are applied over the liquefaction database to create fifteen ensemble models. The performance of ensemble models is compared with single machine learning models, and the effect of data availability on the models’ generalization ability is examined.

## Method

Three ensemble learning frameworks are demonstrated over five single models for the problem of binary classification, namely seismic-induced liquefaction prediction. Bagging, Stacked Generalization and Boosting ensembles are applied. In order to appeal to a wide readership, we use simple notation when referring to the mathematical construct of each ensemble.

Bagging, also known as Bootstrap Aggregation, is one of the most common ensemble learning frameworks^37^. Following the three stages in ensemble learning, we first describe the generation process of the sub-samples. In this learning process, *k* sub-samples are created from the original sample set, *S*, available for training the individual models. Using Bootstrap resampling, each sub-sample, also called a resample *s*_*i*_ (*i* = 1, 2, …, *k*), is generated using random sampling with replacement and has the same size as the original sample. Each observation in the original dataset will have a probability 1/*n* of being chosen, where *n* is the size of the original sample set, *S*. Consequently, some observations may appear more than once in a given subset. The probability that an individual training sample from *S* will not be part of a Bootstrap resampled set is (1 − 1/*n*) ^*n*^ and can be shown to approach 0.37 as the size of *S* increases^38^.

Once the resamples are created, *k* individual machine learning models are generated to carry out the second stage. The type of the individual models is predetermined and their selection can be based on the nature of the problem. Each model will rely on one of the created resamples in order to train and create a relatively unique hypothesis. After all the ensemble members are generated and trained, a unique output for the ensemble is derived by averaging the outputs from these individual models. Suppose that the descriptor variables’ observations of a test instance *i* have been inputted into the *k* individual models; each of these models will have a unique output, or prediction, and the ensemble output is computed using majority voting, as follows:

1 $${\hat{y}}_{i,ensemble}=mode\,({\hat{y}}_{i,1},\,{\hat{y}}_{i,2},\,\ldots ,\,{\hat{y}}_{i,k})$$

where ${\hat{y}}_{ensemble}$ is the resulting ensemble output, and ${\hat{y}}_{i,1}$ is the output from the first individual model, describing the estimate of the *i*^*th*^ test instance. This equation can be used as a combiner for the case of binary classification, as in the current work. If the individual models produce an estimate in a given range between the two classes, as in the case of Logistic Regression, the mean combiner can be used, and the ensemble output is then rounded to produce the final estimate:

2 $${\hat{y}}_{i,ensemble}=round(\frac{1}{k}\mathop{\sum }\limits_{j=1}^{k}{\hat{y}}_{i,j}),\,{\hat{y}}_{i,j}\in [0,1]$$

Because Bagging is essentially a parallel ensemble learning framework, the described algorithm can be parallelized in the computational environment. Furthermore, the main diversity-in-learning manifests from the resampling plan adopted in Bagging. Distinct training data is used to enforce a spectrum of solutions to the individual models, providing improved prediction. The improved generalization ability of Bagging has been discussed and shown over many case studies in the broad literature^33,39,40^.

Stacked Generalization, or Stacking, is an effective way to derive the final ensemble predictions. The linear combination of the outputs of ensemble members is the most popular approach for ensemble combiners^41^. In Stacking, a weighted average that considers the relative performance of each sub-ensemble model is used. Hence, Stacking is an ensemble technique that deals with the ensemble integration particularly^42^. To create the Stacking combiner, an additional model is used to learn how to combine the individual members, by tuning its weights over the feature space. Suppose we derive *k* sub-samples using a particular resampling technique, such as Bootstrapping, and then *k* individual models are created and trained using the generated resamples. The *i*^*th*^ pattern has an observed value *y*_*i*_ and a predicted value, ${\hat{y}}_{i,j}$, obtained from the *j*^*th*^ sub-ensemble model (*j* = *1*, *2*, *…*, *k*). Under Stacking, we label the individual models as level 0 generalizers. At this point, the set of level 0 outputs, for a given pattern, is fed to a level 1 generalizer, which is a separate model that is trained to produce the appropriate output. The common Stacking algorithm suggests minimizing the following error function^43^:

3 $$E({c}_{1},\,{c}_{2},\ldots ,{c}_{k})=\mathop{\sum }\limits_{i=1}^{n}\mathop{\sum }\limits_{j=1}^{k}{[{y}_{i}-{c}_{j}\times {\hat{y}}_{i,j}]}^{2},\,{c}_{j}\ge 0$$

where *y*_*i*_ is the *i*^*th*^ observation from the original training dataset. This algorithm produces estimates, ${\hat{c}}_{j}$, for the combiner coefficients, which are then used to construct the ensemble prediction as follows:

4 $${\hat{y}}_{i,ensemble}=round(\mathop{\sum }\limits_{j=1}^{k}{c}_{j}\times {\hat{y}}_{i,j}),\,{\hat{y}}_{i,j}\in [0,1]$$

It is necessary to highlight the need for nonnegative coefficients which lead to an improved generalization ability of the bias-variance decomposition of Stacked ensemble models investigated^33^. Moreover, equation (3) minimizes the sum of squared differences between observed and predicted values. When used to determine the coefficients, this process may be dominated by those patterns with a large error. A better choice, as adopted in this work, is to minimize the (squared) relative difference. The objective function for the relative difference is constructed as follows:

5 $$RE({c}_{1},\,{c}_{2},\,\ldots ,\,{c}_{k})=\mathop{\sum }\limits_{i=1}^{n}\mathop{\sum }\limits_{j=1}^{k}{[\frac{{y}_{i}-{c}_{j}\times {\hat{y}}_{i,j}}{{y}_{i}}]}^{2},\,{c}_{j}\ge 0$$

Solutions to the generalized Stacking coefficients are then used in the model. In this work, we further modify equation (5) to have a normalized weighted sum constraint $\mathop{\sum }\limits_{j=1}^{k}{c}_{j}=1$, and use the final ensemble combiner. Using this constraint in binary classification problems is justified, and it is expected to drive further improvement in the overall ensemble performance, as the effect of the normalized coefficients can be observed in the bias-variance-covariance decomposition of the ensemble’s error function.

Boosting is an in-series ensemble learning framework for any given set of single machine learning models. In every training step, a reweighted version of the original training set is used based on the model performance over the feature space. Boosting ensemble learning has undergone intense theoretical studies and empirical testing^44,45^. There are several Boosting versions in the literature, including AdaBoost, AdaBoost.M1, AdaBoost.M2 and AdaBoost.R^46^. Moreover, the AdaBoost ensemble model is for binary classification problems and will be used in the current study as one of the investigated ensembles.

The considered Boosting ensemble starts with one weak learner and trains it with equally likely observations. In other words, the resample used to train the first weak learner comes from a random sampling with replacement, where all the observation in the original dataset have equal weights (probability of sampling), such as:

6 $${w}_{1,i}=\frac{1}{n},\,i=1,\,2,\,3,\,\ldots ,\,n$$

where *w*_*l*, *i*_ is the first-stage weight of the *i*^*th*^ training observation. Once the model is trained, all the available observations are estimated. The error function for the *j*^th^ sub-model, used for the binary classification problem, is formulated as follows:

7 $${E}_{j}=\frac{1}{n}\mathop{\sum }\limits_{i=1}^{n}{w}_{j,i}\times I[{y}_{i}\ne round({\hat{y}}_{i,j})]$$

where *I* is the identity operator, returning 1 when the enclosed condition is satisfied and 0 otherwise. The error function is simply the probability of misclassifying an observation by the individual model. Hence, based on the estimation error, the data weights are updated. In a classification setting, the observations which are incorrectly classified will have larger weights and vice versa. In addition, this learner will have a collective weight which is associated with its overall performance. In other words, the *j*^*th*^ learner’s performance measure is formulated as follows:

8 $${a}_{j}=\frac{1}{2}\times \,\mathrm{log}(\frac{1-{E}_{j}}{{E}_{j}})$$

The updated weights are then calculated based on the following piecewise function:

9 $${w}_{j,i}=\{\begin{array}{ll}{w}_{j-1,i}\times {e}^{-{a}_{j}}, & {y}_{i}=round({\hat{y}}_{i,j-1})\\ {w}_{j-1,i}\times {e}^{{a}_{j}}, & {y}_{i}\ne round({\hat{y}}_{i,j-1})\end{array}$$

This process is then repeated for all the sub-models. As a consequence of the weight-updating and performance-measuring process, the next sub-model will attempt to fix the errors made by the previous learner. The following section provides more details on the utilization of the considered ensemble approaches to construct ensemble classification models for liquefaction occurrence over the case study.

## Results and Discussion

### The performance of the ensemble models over the case study

The focus of the current study is to demonstrate the application of ensemble learning approaches for liquefaction prediction. Five different single models are considered in this study. Hence, for each ensemble architecture, an ensemble model is created using one of these single models. More precisely, Ensemble-based Logistic Regression (ELR), Support Vector Machines (ESVM), Decision Trees, Extreme Learning Machine (EELM) and Artificial Neural Network (EANN) models are considered. Ensemble models of Decision Trees are commonly known in the broad literature as Random Forests (RF).

An optimal configuration of the single Artificial Neural Networks and Extreme Learning Machines should be decided before the ensemble model is created. For example, a cross-validation study is carried out to determine the number of hidden layers and hidden neurons for individual ANNs. In this study, a feedforward multi-layer perceptron ANN with one hidden layer and eight hidden neurons is considered. This configuration is optimum for the current case study^10,47^. The log-transform function is used as the hidden neurons’ transfer function^18^. Moreover, Levenberg-Marquardt (LM) algorithm is used to train the individual ANNs. In the case of Extreme Learning Machine (ELM) models, the training follows a recently recommended approach in the literature^48^. Also, the utilized kernel for individual SVMs is the Radial Basis Function^18^. The Bayesian optimization approach is used to solve for the SVM’s optimal configuration^49^. There are different approaches to ensure sufficient regularization of single models. In the present work, different regularization techniques are applied to meet the individual models, simulation cost and available information requirements. For the case of LRs and pruned Decision Trees, the cross validation of the individual models, based on the training set within a Monte Carlo simulation instance, is used to regularize the sub-ensembles. Regularization of the ANNs is enforced by the early stopping procedure. Finally, the regularization of the utilized ELMs and SVMs is enforced through their Bayesian regularization based training algorithms.

The database used in this study has been originally compiled in the literature^50^. Earthquake observations from 85 sites are available in the final database, where 42 sites have experienced liquefaction. Eight variables are considered as explanatories to liquefaction potential (Table 1). Earthquake magnitude (*M*), total vertical stress (*σ*_*o*_), effective vertical stress (*σ*_*o*_’), standardized SPT (*N*_1_)_60_, normalized peak horizontal acceleration (*a/g*), equivalent dynamic shear stress (***τ***_***av***_/***σ***_***o***_***’***), fines content (*F*), and the average grain size (*D*_50_), are used in this study. The significance of the utilized database have been thoroughly investigated in the relevant literature for liquefaction assessment^47,51^. While this particular database is used in the current study, many other databases exist in the literature and can be used. However, a preliminary analysis should be carried out in order to determine the optimum explanatory variables from the available database as well as determine a class-balancing procedure in case the database has a relatively large difference in the number of observations for each class^44^. In this work, a feature’s relative importance test is carried out for each of the individual models (Supplementary Table S1 as well as Supplementary Fig. 1). The test is based on the *Kappa* statistic, or Cohen’s Kappa coefficient (***κ***)^52^, where the complement of the drop in the performance of the model due to omitting a feature is defined as that feature’s relative importance^53^. The obtained results shows that each feature’s relative performance, while varying among different learners, is above 6.25%, which is the threshold of considering a features addition to the set of explanatories. In addition, (*N*_1_)_60_ is shown to have the highest relative importance, which is reported in previous work as the most important variable to explaining liquefaction occurrence^9,50^.

**Table 1:** Descriptive statistics of the study variables.

Variable	Unit	Minimum	Mean	Median	Max	Stand. Dev.
**Training dataset**						
*M*	Richter	5.50	7.33	7.50	8.30	0.58
*σ*_*o*_	kPa	50.00	110.62	93.20	686.70	83.88
*σ*_*o*_′	kPa	28.40	63.26	63.80	105.90	20.03
*(N*_*1*_ *)*_*60*_	—	1.00	10.77	9.00	31.00	6.86
*a/g*	—	0.10	0.22	0.19	0.60	0.12
*τ*_*av*_/*σ*_*o*_′	—	0.08	0.21	0.17	0.45	0.11
*F*	(%)	0.00	9.05	5.00	35.00	9.61
*D*_*50*_	mm	0.09	0.39	0.30	1.60	0.30
**Testing dataset**						
*M*	Richter	6.10	7.30	7.40	7.40	0.35
*σ*_*o*_	kPa	59.80	99.94	100.05	247.20	37.84
*σ*_*o*_′	kPa	34.30	64.44	66.70	105.90	18.04
*(N*_*1*_ *)*_*60*_	—	4.00	11.27	10.00	23.00	5.60
*a/g*	—	0.10	0.24	0.24	0.32	0.05
*τ*_*av*_/*σ*_*o*_′	—	0.09	0.21	0.21	0.35	0.06
*F*	(%)	0.00	8.85	8.50	27.00	7.33
*D*_*50*_	mm	0.12	0.42	0.35	1.60	0.33

Moreover, training and testing sets are usually designed to represent similar characteristics, the nature of the problem incurs constructing a testing set from sites different than the training set in order to test the model’s capability in the regional prediction problem. Due to the regional diversity of the sites, testing and training sets may have slight differences in some of the features’ characteristics, as seen in *σ*_*o*_. In the present study, the selection of the testing set as seismic events from completely different sites is important in order to test the model’s capability in the regional prediction problem. This decision is critical to report reliable testing performance, given the nature of the application. The latter explains the variation in the descriptive statistics reported in Table 1.

The selection and processing of the study features and labeling of events follows similar work in the literature^47^. More specifically, the final database consists of 73 sites from Japan and 12 sites from the United States and Pan-America. The output (class) is binary-type which takes the value of 1 for sites apparent liquefaction, and a value of 0 otherwise. Incomplete records, from the original set, or records with Fines content greater than 35% are not considered in the reported final dataset. Observations with relatively high Fines content are omitted because of the unreliability of generalizing trends from the unavailability of data in that range. Preprocessing of the field data involved normalizing the features such that the minimum and maximum observations, pear feature, is set between 0 and 1. The experimental setup and simulations are carried out in MATLAB environment.

Machine learning models are typically instable. Multiple training attempts of machine learning models using the same training observation and optimization technique may not produce the same solution; this is due to the relatively higher nonlinear formulation of such models, which prompt local optimality in the solution of their parameter^54,55^. Consequently, in assessing the performance of a machine learning model, a Monte Carlo simulation should be carried out and the average performance of the simulation should be reported for a more reliable assessment. Each training and testing Monte Carlo simulation is generated by a complete ensemble model creation, training and testing. Table 2 presents the average training and testing performance of the fifteen developed ensemble models as well as the single models. The table also reports the change in the training performance with respect to the increase in ensemble size. The ensemble models are run for ensemble sizes from 5 to 50. The same experiments are used to generate the corresponding ***κ***–based results (Supplementary Table S2).

**Table 2:** Average training (top) and testing (bottom) results of the ensemble models with varying ensemble size.

Model\Size	1	5	10	15	20	25	30	40	50
***Accuracy*** **(Training)**									
ELR - Bagging	0.9142	0.9576	0.9614	0.9664	0.9693	0.9714	0.9700	0.9719	0.9739
ELR - Stacking		0.9631	0.9761	0.9810	0.9842	0.9841	0.9861	0.9873	0.9880
ELR - Boosting		0.9995	1.0000	1.0000	1.0000	1.0000	1.0000	1.0000	1.0000
ESVM- Bagging	0.8171	0.8664	0.8695	0.8720	0.8747	0.8747	0.8768	0.8759	0.8741
ESVM - Stacking		0.8859	0.9024	0.9141	0.9202	0.9220	0.9197	0.9271	0.9300
ESVM - Boosting		0.8973	0.9412	0.9700	0.9793	0.9883	0.9929	0.9968	0.9988
RF - Bagging	0.8771	0.9439	0.9549	0.9608	0.9651	0.9676	0.9680	0.9719	0.9702
RF - Stacking		0.9541	0.9758	0.9792	0.9864	0.9895	0.9927	0.9949	0.9958
RF - Boosting		0.9978	1.0000	1.0000	1.0000	1.0000	1.0000	1.0000	1.0000
EELM - Bagging	1.0000	1.0000	1.0000	1.0000	1.0000	1.0000	1.0000	1.0000	1.0000
EELM - Stacking		1.0000	1.0000	1.0000	1.0000	1.0000	1.0000	1.0000	1.0000
EELM - Boosting		0.9980	0.9998	1.0000	1.0000	1.0000	1.0000	1.0000	1.0000
EANN - Bagging	0.8690	0.9508	0.9631	0.9758	0.9792	0.9822	0.9831	0.9817	0.9793
EANN - Stacking		0.9625	0.9849	0.9920	0.9954	0.9966	0.9973	0.9988	0.9997
EANN - Boosting		0.9927	1.0000	1.0000	1.0000	1.0000	1.0000	1.0000	1.0000
***Accuracy*** **(Testing)**									
ELR - Bagging	0.8204	0.8673	0.8754	0.8831	0.8827	0.8842	0.8804	0.8842	0.8850
ELR - Stacking		0.8619	0.8673	0.8788	0.8746	0.8746	0.8688	0.8777	0.8800
ELR - Boosting		0.8469	0.8635	0.8688	0.8796	0.8785	0.8781	0.8850	0.8804
ESVM- Bagging	0.8050	0.8569	0.8662	0.8804	0.8715	0.8785	0.8738	0.8781	0.8785
ESVM - Stacking		0.8531	0.8523	0.8596	0.8554	0.8662	0.8688	0.8581	0.8662
ESVM - Boosting		0.8465	0.8596	0.8477	0.8542	0.8673	0.8673	0.8696	0.8704
RF - Bagging	0.6458	0.6846	0.7108	0.6908	0.7192	0.7069	0.7146	0.7200	0.7212
RF - Stacking		0.6808	0.6992	0.6788	0.7131	0.7158	0.7219	0.7196	0.7181
RF - Boosting		0.7162	0.7396	0.7515	0.7538	0.7692	0.7627	0.7685	0.7712
EELM - Bagging	0.6846	0.6992	0.7108	0.7031	0.7065	0.7065	0.7058	0.7019	0.7042
EELM - Stacking		0.6992	0.7015	0.7031	0.7031	0.7065	0.7023	0.6996	0.7035
EELM - Boosting		0.6646	0.6692	0.6662	0.6631	0.6612	0.6665	0.6654	0.6662
EANN - Bagging	0.7415	0.8231	0.8423	0.8669	0.8577	0.8750	0.8719	0.8654	0.8688
EANN - Stacking		0.8219	0.8446	0.8488	0.8535	0.8392	0.8600	0.8569	0.8565
EANN - Boosting		0.8273	0.8662	0.8762	0.8800	0.8796	0.8854	0.8904	0.8965

From the obtained training performance results, it is clear that ensemble models, even with the smallest ensemble size, significantly outperform single models. With the increasing ensemble size, the models’ training performance gradually increases to reach perfect classification ability. In fact, except for ESVMs, Boosted ensembles reach 100% classification *Accuracy* (1.0 ***κ***) starting from ensembles of size 10. Bagging and Stacking ensembles sustain the gradual improvement in classification performance with respect to the increase in the ensemble size. Furthermore, the EELM models, over all ensemble architectures, overfit to the training data starting from the smallest ensembles. This is due to the adopted learning strategy of the individual EELM models. Such saturation in training performance is misleading and requires an additional set of estimates; hence, the testing set is required for a complete reliable performance assessment. From the testing results, it can be observed that no model achieves a perfect classification performance, which is expected and should most likely be the case in any classification problem.

The testing performance, however, has the same gradual increase with respect to the increase in ensemble size. Also, the performance of the ensembles is better compared with single models. Boosted EANNs of ensemble size 50 have the best average testing results over the two performance evaluation criteria. On the other hand, all types of EELM models produce the worst testing performance. This result confirms the necessity of a third dataset (testing set) to evaluate the models, as EELMs have shown perfect classification performance in the training stage. It is also interesting to observe that ELR models produce the second-best performance over the testing set. This result confirms the ability of ensembles to significantly increase the performance of relatively simpler single models and, in return, provide robust combination of classically preferred linear models. However, it should be noted that such result is case-dependant and different case studies may show different performance by the simple models.

**Figure 3:**
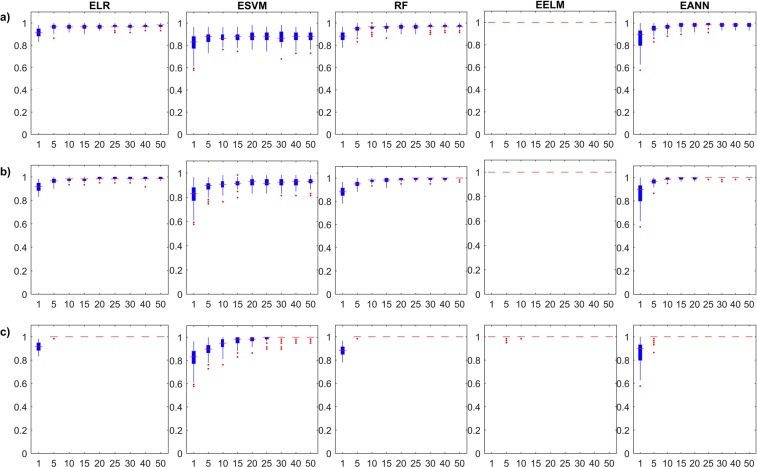
Monte Carlo simulation of the investigated ensemble models’ training *Accuracy* results with respect to ensemble size; (**a**) Bagging models, (**b**) Stacking models, and (**c**) Boosting models.

As mentioned earlier, the stability of the investigated models is an important aspect that should be investigated in the comprehensive analysis of machine learning models. Hence, Fig. 3 depicts the boxplots of the Monte Carlo simulations in the training stage of the single and ensemble models in terms of *Accuracy*. In addition, the ***κ***–based results are presented in Supplementary Fig. 2. In addition, rows (a), (b), and (c) group models with the same ensemble framework; row (a) shows the performance of Bagged ensembles, while rows (b) and (c) show the Stacked and Boosted ensembles, respectively.

**Figure 4:**
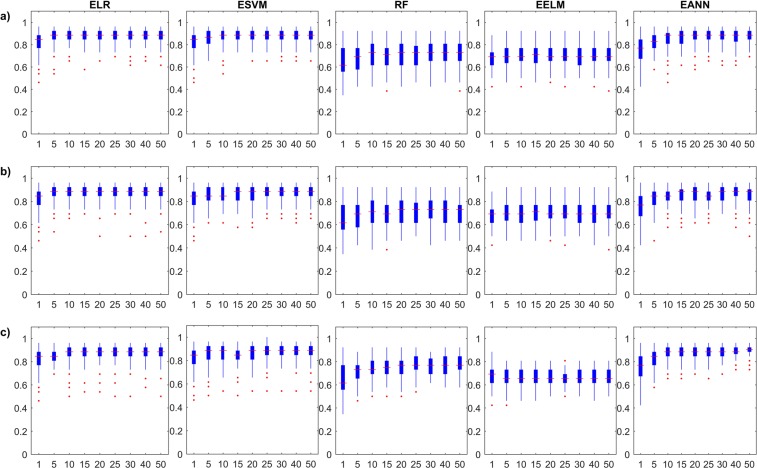
Monte Carlo simulation of the investigated ensemble models’ testing *Accuracy* results with respect to ensemble size; (**a**) Bagging models, (**b**) Stacking models, and (**c**) Boosting models.

As expected from the results on the training stages, the boxplots return little information on the reliability of the investigated models, especially those for EELM models. It is interesting to observe that *Accuracy* and ***κ***–based Monte Carlo simulations for both ELR and ESVM models show slight decrease in the training performance with increasing ensemble size, but with tighter confidence. This is attributed to the curse of dimensionality which linear learners (with linear combiners) may suffer from as the number of sub-ensemble estimates increase. In general, the uncertainty in the learners matches the expected behaviour of diminishing with the increase in the ensemble size. Moreover, Fig. 4 and Supplementary Fig. 3 depict the boxplots of the Monte Carlo simulations in the testing stage of the single and ensemble models in terms of the *Accuracy* and ***κ***, respectively. The testing boxplots of the five different single models used in this study show a variable behaviour from different aspects, which demonstrate the importance of this comprehensive study. ***κ***–based results are proportionate in testing, for each ensemble model. For example, when looking at the performance of EELMs, we can see that they have very little improvement with increase in ensemble size and are poor in generalization description (if only training performance is reported). When examining EANNs, the typical increase in the model performance as the ensemble size grows is observed. Also, the stability in the model performance substantially increases, similar to what has been reported in the general literature. The inspection of the five single models shows that ELRs, ESVMs and EANNs are the most stable models with diminishing uncertainties.

Boosting and Bagging strategies produce the best, and second-best, performance over all the models, respectively. Stacked Generalization performance falls behind. The authors attribute this issue to the effect of increased ensemble size on the reliability of the second-level learner that trains the linear combination parameters in Stacking. As discussed earlier, the curse of dimensionality arises from the increased size of the ensemble model. The reader may attempt to attribute this effect on Boosted models as they also produce linear combining parameters. However, the nature of the Boosting-based parameters is related to the model performance and data sampling rather than regression-based fitting of the models’ outputs. The parameters in the Boosting framework allow for value explosion in a binary classification setting (i.e., the ensemble estimates can exceed the −1/1 boundaries by a very large difference). This parameter nature requires a release from the curse of dimensionality in higher ensemble sizes for Boosted models. The Bagged ensembles are then placed second-best as they do not attempt a targeted preference among the ensemble member estimates.

### Performance with respect to data availability

**Figure 5:**
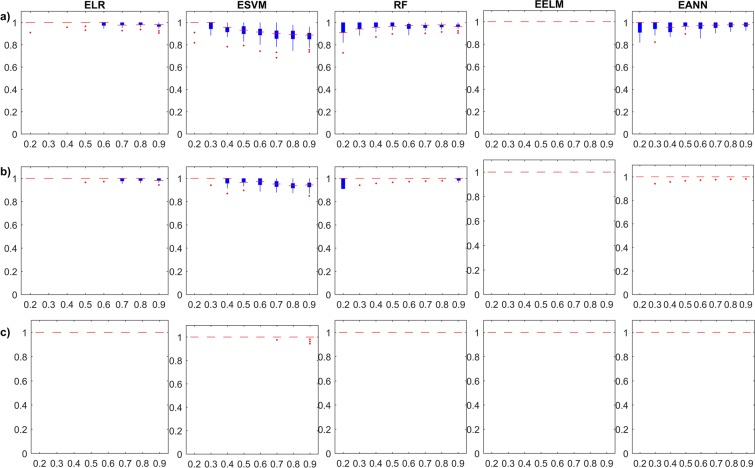
Monte Carlo simulation of the investigated ensemble models’ training *Accuracy* results with respect to data availability; (**a**) Bagging models, (**b**) Stacking models, and (**c**) Boosting models.

**Figure 6:**
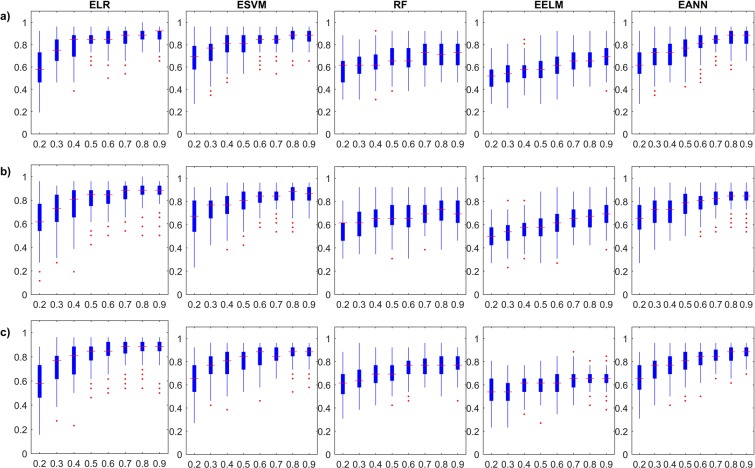
Monte Carlo simulation of the investigated ensemble models’ testing *Accuracy* results with respect to data availability; (**a**) Bagging models, (**b**) Stacking models, and (**c**) Boosting models.

A final evaluation should be carried out over the ensembles’ performances with respect to limited availability of training data. This analysis facilitates a decision on the adequacy and sufficiency of the total available dataset. Table 3 and Supplementary Table S3 show the training and testing performance of the investigated ensemble models when a portion of the training data is randomly selected and used for the training of the ensemble members and the ensemble integration techniques (for Stacking and Boosting). In this analysis, the ensemble size is fixed to 50 which is the highest in the previous analysis. Figures 5 and 6 present the Monte Carlo training and testing simulations for this analysis, respectively. The corresponding ***κ***–based results are presented in Supplementary Figs 4 and 5. The selected proportions vary from 20% to 90% throughout all the ensembles. All other factors are fixed in this analysis to observe the effect of data availability on performance. The Monte Carlo simulation is carried out for each proportion case such that in each simulation the partitioned training data is resampled again but with the same ratio with respect to the available dataset.

**Table 3:** Average training (top) and testing (bottom) results of the ensemble models with varying data availability.

Model	20%	30%	40%	50%	60%	70%	80%	90%
***Accuracy*** **(Training)**								
ELR - Bagging	0.9991	1.0000	0.9987	0.9959	0.9880	0.9829	0.9796	0.9755
ELR - Stacking	1.0000	1.0000	1.0000	0.9983	0.9969	0.9907	0.9913	0.9887
ELR - Boosting	1.0000	1.0000	1.0000	1.0000	1.0000	1.0000	1.0000	1.0000
ESVM- Bagging	0.9882	0.9647	0.9396	0.9228	0.9066	0.8920	0.8851	0.8808
ESVM - Stacking	1.0000	0.9959	0.9822	0.9703	0.9663	0.9502	0.9411	0.9383
ESVM - Boosting	1.0000	1.0000	1.0000	1.0000	1.0000	0.9998	1.0000	0.9970
RF - Bagging	0.9373	0.9535	0.9661	0.9679	0.9657	0.9678	0.9681	0.9700
RF - Stacking	0.9764	0.9935	0.9965	0.9952	0.9963	0.9963	0.9962	0.9943
RF - Boosting	1.0000	1.0000	1.0000	1.0000	1.0000	1.0000	1.0000	1.0000
EELM - Bagging	1.0000	1.0000	1.0000	1.0000	1.0000	1.0000	1.0000	1.0000
EELM - Stacking	1.0000	1.0000	1.0000	1.0000	1.0000	1.0000	1.0000	1.0000
EELM - Boosting	1.0000	1.0000	1.0000	1.0000	1.0000	1.0000	1.0000	1.0000
EANN - Bagging	0.9718	0.9653	0.9622	0.9724	0.9697	0.9773	0.9762	0.9777
EANN - Stacking	1.0000	0.9994	0.9987	0.9997	0.9994	0.9995	0.9996	0.9992
EANN - Boosting	1.0000	1.0000	1.0000	1.0000	1.0000	1.0000	1.0000	1.0000
***Accuracy*** **(Testing)**								
ELR - Bagging	0.5800	0.7435	0.7915	0.8335	0.8465	0.8631	0.8765	0.8808
ELR - Stacking	0.6254	0.7169	0.7715	0.8019	0.8242	0.8504	0.8665	0.8727
ELR - Boosting	0.5850	0.7173	0.7735	0.8146	0.8338	0.8565	0.8646	0.8746
ESVM- Bagging	0.6750	0.7292	0.7896	0.8012	0.8388	0.8385	0.8735	0.8627
ESVM - Stacking	0.6708	0.7369	0.7627	0.7981	0.8242	0.8438	0.8492	0.8573
ESVM - Boosting	0.6654	0.7492	0.7727	0.8146	0.8400	0.8500	0.8635	0.8669
RF - Bagging	0.5904	0.6123	0.6377	0.6719	0.6612	0.7104	0.7119	0.7119
RF - Stacking	0.5650	0.6165	0.6508	0.6750	0.6638	0.6958	0.7100	0.7050
RF - Boosting	0.6046	0.6581	0.6865	0.7092	0.7381	0.7596	0.7650	0.7769
EELM - Bagging	0.5150	0.5362	0.5631	0.5735	0.6042	0.6542	0.6681	0.6846
EELM - Stacking	0.5119	0.5331	0.5623	0.5731	0.6035	0.6531	0.6665	0.6800
EELM - Boosting	0.5404	0.5381	0.5827	0.5938	0.6135	0.6338	0.6473	0.6577
EANN - Bagging	0.6342	0.6935	0.7281	0.7550	0.7985	0.8346	0.8550	0.8635
EANN - Stacking	0.6596	0.7062	0.7242	0.7669	0.8004	0.8150	0.8385	0.8396
EANN - Boosting	0.6573	0.7335	0.7638	0.7942	0.8277	0.8542	0.8627	0.8715

It is interesting to observe contradicting performance behavior between the training and the testing datasets, which is also expected in the proportional data case. For example, the training results of the Bagged, Stacked and Boosted ESVM models decrease with the increase of training data availability. This behavior is also captured in the remaining ensemble models, except for EELM due to the reasons mentioned before. In addition, the results of Boosted models do not properly show this behavior as the two other ensemble approaches. On the other hand, the testing results clearly show the increasing *Accuracy* with the increase of training data availability. As it is generally accepted that more information produces more generalized learners, the stability (diminishing uncertainty) and increased generalization ability are shown.

It is also important to note that close inspection of the testing results reveals marginal improvement in the model generalization ability before data saturation. For example, Bagging-type RF models show the best testing results at 70% data proportion. Although this result is only the best among RF models and is only shown in limited number of models, they have a conceptual meaning. The diversity manifesting from the ensemble learning itself, which is the main contributor to the improved performance, is partly driven by the unique information fed to the sub-ensembles. The reason behind the absence of abundant observations (Bagged RF at 70% data availability) in the simulation results among all ensembles is strongly related to the nature of the ensemble model itself. In fact, this result is the motivation for state-of-the-art ensemble frameworks that utilize the diversity concept more explicitly in their architecture^30,33^. The explicit utilization of diversity concept is expected to provide further improvement to capture the patterns usually weaker than the ones on which hyperplanes are based.

## Conclusion

Ensemble learning offers much needed solutions for direct classification of seismic-induced liquefaction, which are not usually obtained by single machine learning models, due to instability, nor by deep learning models, due to data\feature limitation. The cross-sectional investigation of the various ensemble learning frameworks is needed in such problem, but is usually overlooked. Hence, this work aims at motivating the development of state-of-the-art ensemble approaches for regional liquefaction prediction. Three ensemble learning frameworks are utilized with five different machine learning models. The added benefit of ensemble learning is demonstrated through the various targeted testing schemes. A Monte Carlo simulation is carried out for each ensemble model in order to further investigate the improved generalization ability of ensemble learning. In terms of experimental setup, the work provides recommendations to evaluate the data availability challenge when developing ensemble models to the problem of interest, which is a major challenge in this field. On the specific ensemble learning level, this paper presents the application of Stacking based ensembles, which has not been studied for liquefaction prediction in the literature. On the sub-ensemble level, this paper presents the results of Boosted and Bagged models based on previously unexamined individual learners’ pairing (within the ensemble learning application). More research is required to adequately address ensemble learning in this field. The explicit utilization of diversity concept in developing ensemble learning models is expected to provide further improvement to the prediction. Such approach is expected to capture the patterns usually hidden or weaker than the ones on which hyperplanes are simulated by the previous models.

## Supplementary information

Supplementary Information

Dataset 1

Dataset 2

Dataset 3

Dataset 4

Dataset 5

Dataset 6

Dataset 7
