# Supplementary material for: Predicting seismic-induced liquefaction through ensemble learning frameworks
Source: Sci Rep. 2019 Aug 13;9:11786. doi: 10.1038/s41598-019-48044-0 (PMC6692379; doi:10.1038/s41598-019-48044-0)
Supplement: Supplementary file 1 — Supplementary Information [file 41598_2019_48044_MOESM1_ESM.pdf]

Supplementary information for

Predicting seismic-induced liquefaction through ensemble learning frameworks

Mohammad H. Alobaidi[1,*], Mohamed A. Meguid[1], Fateh Chebana[2]

[1] Department of Civil Engineering and Applied Mechanics, McGill University, 817 Sherbrooke Street West, Montréal (QC), H3A 2K6, Canada

[2] Eau Terre Environnement, Institut National de la Recherche Scientifique, 490 Rue de la Couronne, Québec (QC), G1K 9A9, Canada

[*]Corresponding author;
Email: mohammad.alobaidi@mcgill.ca
Tel: +1 514-398-1609

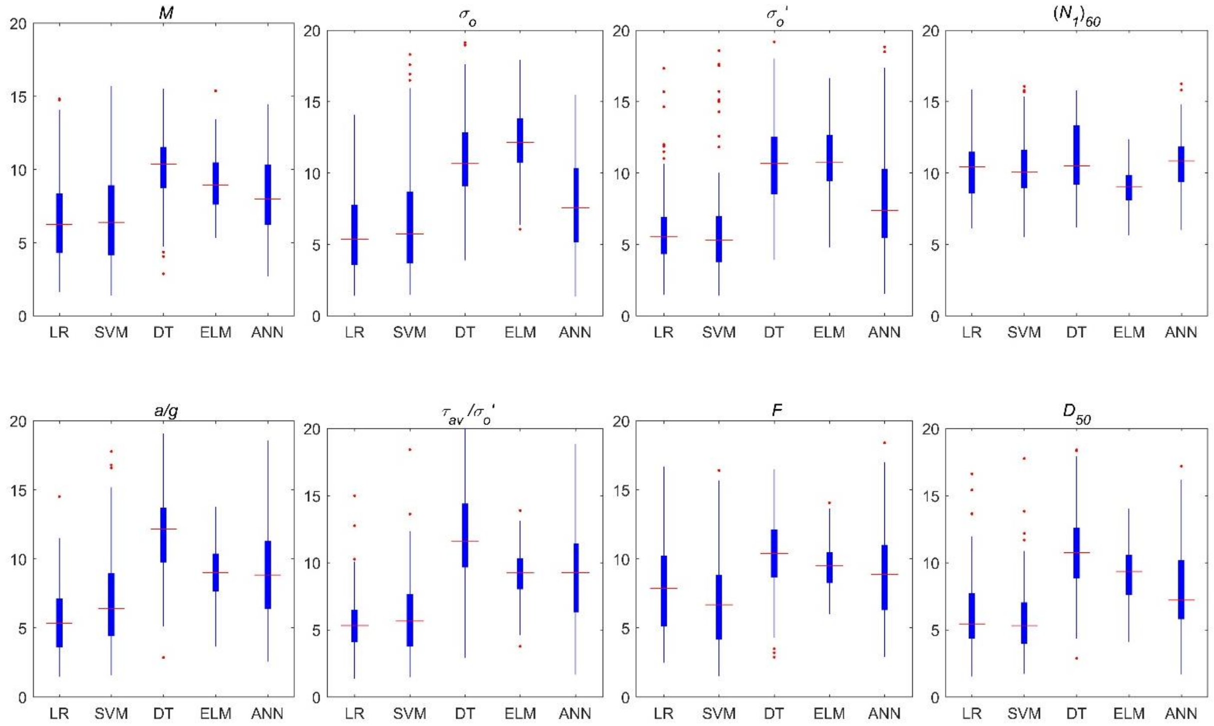

Supplementary Figure 1. Monte Carlo simulation of features' relative importance for each individual model.

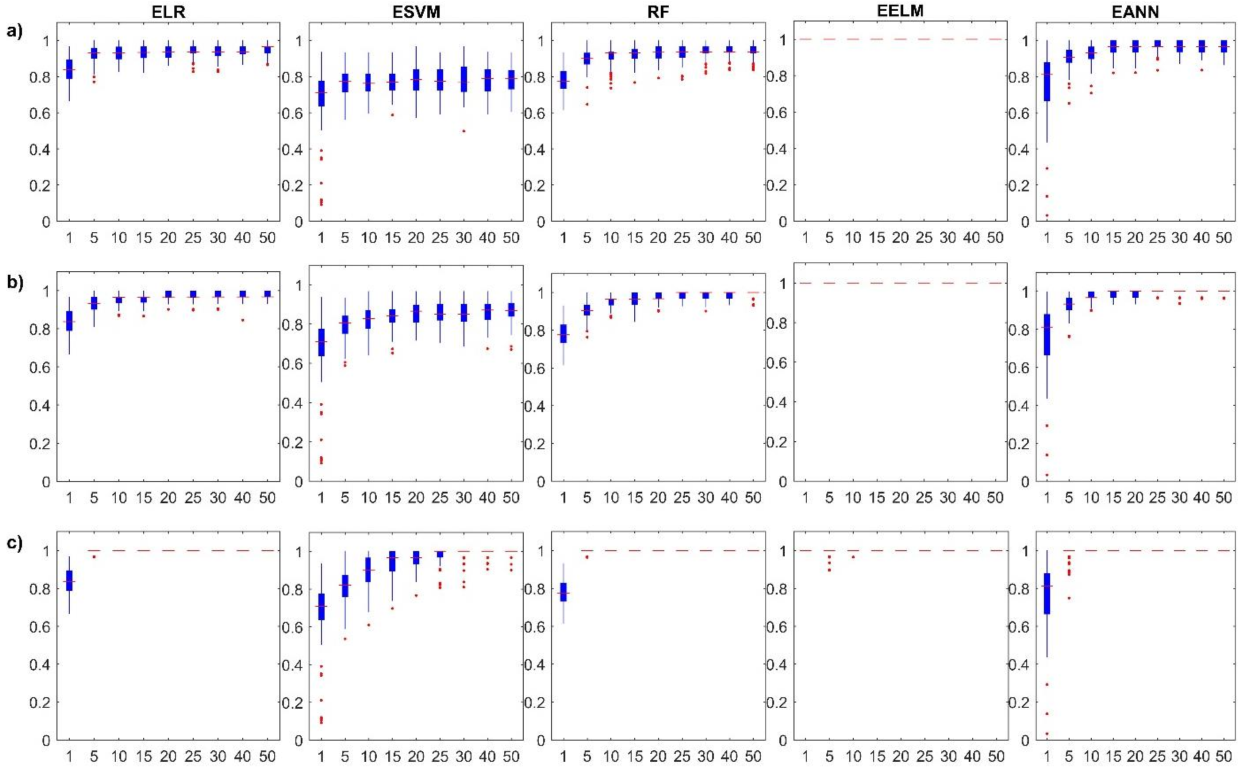

Supplementary Figure 2. Monte Carlo simulation of the investigated ensemble models' training *Kappa* results with respect to ensemble size; a) Bagging models, b) Stacking models, and c) Boosting models.

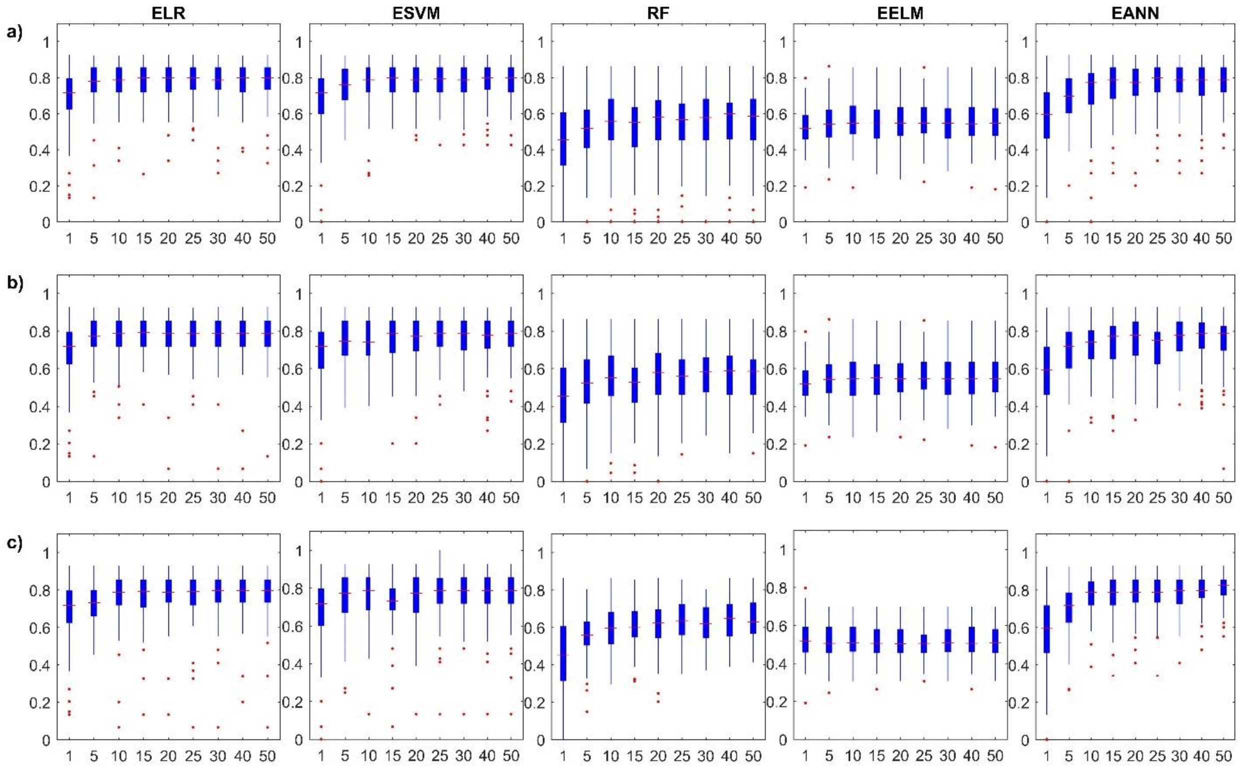

Supplementary Figure 3. Monte Carlo simulation of the investigated ensemble models' testing *Kappa* results with respect to ensemble size; a) Bagging models, b) Stacking models, and c) Boosting models.

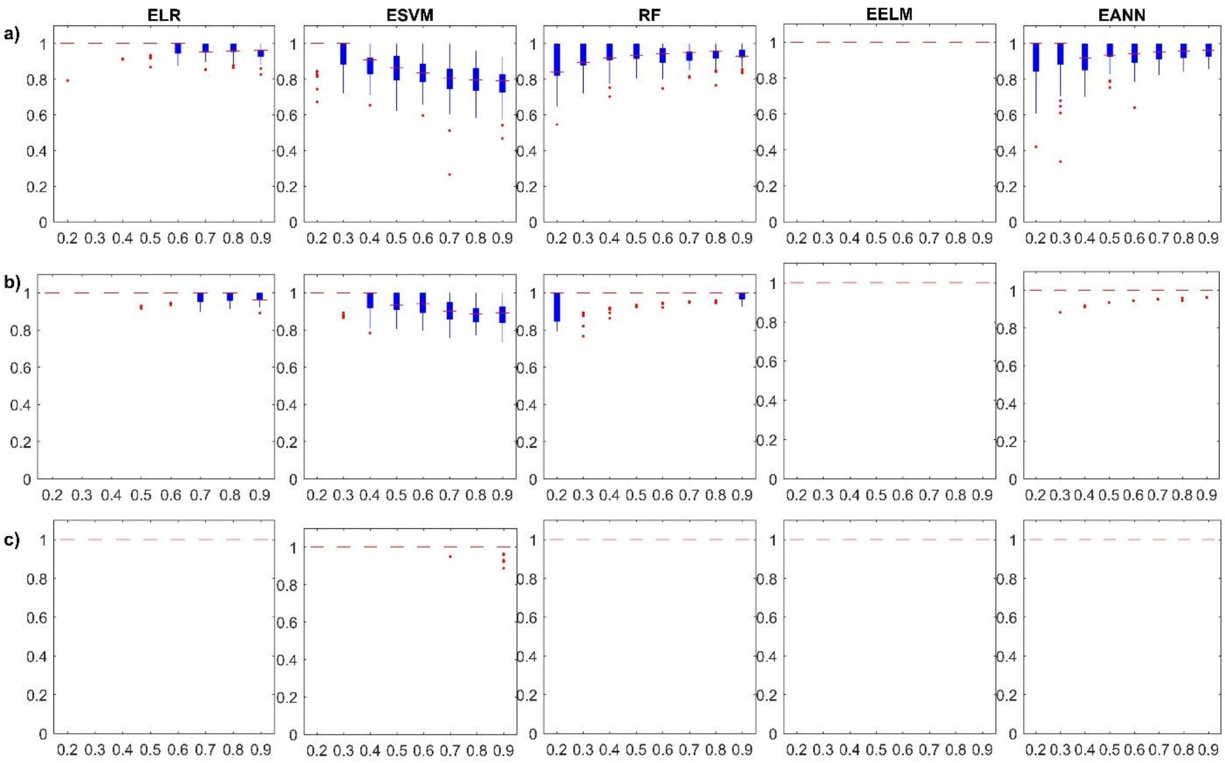

Supplementary Figure 4. Monte Carlo simulation of the investigated ensemble models' training *Kappa* results with respect to data availability; a) Bagging models, b) Stacking models, and c) Boosting models.

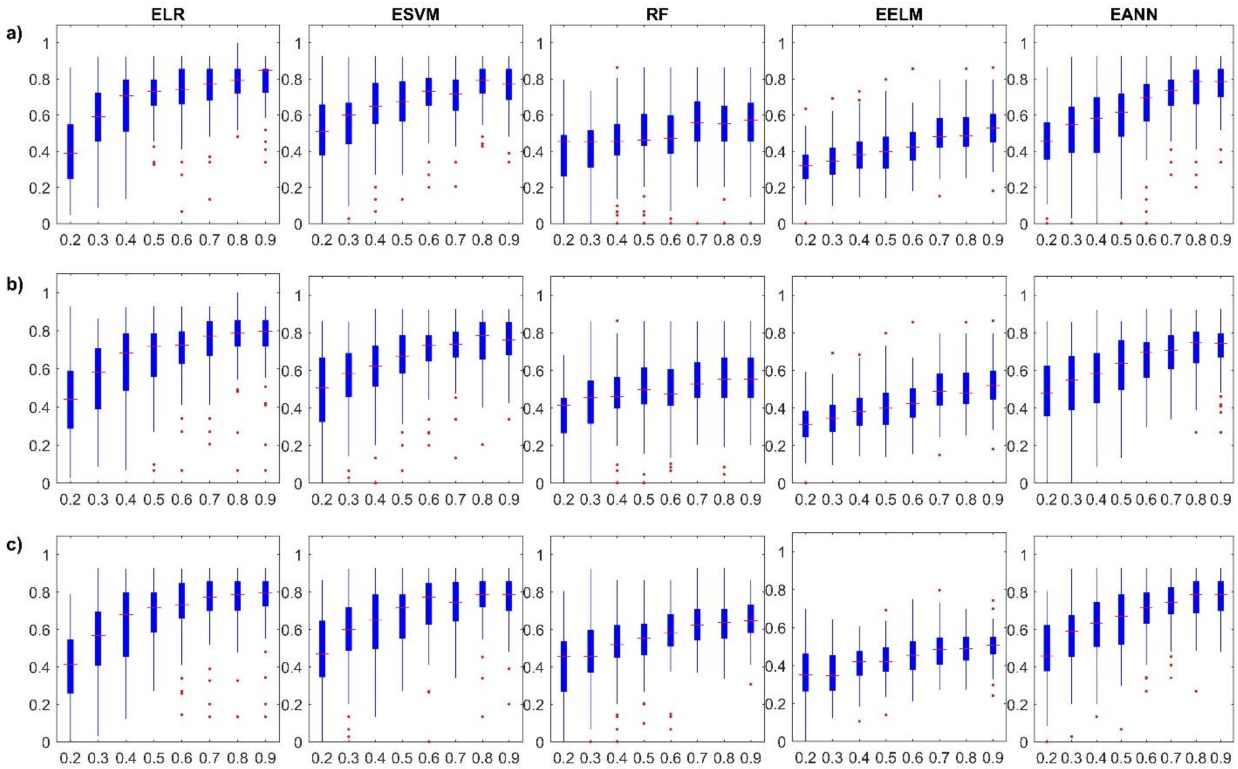

Supplementary Figure 5. Monte Carlo simulation of the investigated ensemble models' testing *Kappa* results with respect to data availability; a) Bagging models, b) Stacking models, and c) Boosting models.

Supplementary Table S1. Average relative Importance (rank) of the study variables for each of the individual models.

Variable \ Model	LR	SVM	DT	ELM	ANN
M	11.72 (3)	11.41 (5)	10.87 (8)	11.07 (8)	11.03 (7)
σ_o	10.47 (6)	12.36 (2)	12.65 (4)	15.60 (**1**)	11.08 (6)
σ_o'	10.69 (5)	10.41 (6)	11.87 (6)	13.69 (2)	11.32 (5)
$(N_1)_{60}$	21.47 (**1**)	21.29 (**1**)	14.71 (**1**)	13.41 (3)	17.73 (**1**)
a/g	10.36 (7)	12.32 (3)	13.20 (2)	11.34 (6)	12.50 (4)
τ_{av} / σ_o'	09.54 (8)	10.19 (7)	12.91 (3)	11.48 (5)	12.70 (3)
F	14.94 (2)	12.10 (4)	12.07 (5)	12.21 (4)	12.92 (2)
D_{50}	10.80 (4)	09.92 (8)	11.71 (7)	11.21 (7)	10.72 (8)

Supplementary Table S2. Average training (top) and testing (bottom) results of the ensemble models with varying ensemble size.

κ (Training)									
**Model \ Size**	**1**	**5**	**10**	**15**	**20**	**25**	**30**	**40**	**50**
**ELR - Bagging**		0.9172	0.9230	0.9339	0.9387	0.9434	0.9403	0.9439	0.9481
**ELR - Stacking**	0.8368	0.9267	0.9525	0.9621	0.9682	0.9678	0.9720	0.9744	0.9756
**ELR - Boosting**		0.9990	1.0000	1.0000	1.0000	1.0000	1.0000	1.0000	1.0000
**ESVM- Bagging**		0.7639	0.7666	0.7731	0.7762	0.7792	0.7819	0.7815	0.7781
**ESVM - Stacking**	0.6725	0.7940	0.8223	0.8412	0.8512	0.8539	0.8507	0.8632	0.8676
**ESVM - Boosting**		0.8144	0.8915	0.9422	0.9600	0.9767	0.9859	0.9937	0.9976
**RF - Bagging**		0.8921	0.9110	0.9238	0.9321	0.9366	0.9372	0.9450	0.9419
**RF - Stacking**	0.7764	0.9108	0.9523	0.9588	0.9731	0.9791	0.9855	0.9899	0.9916
**RF - Boosting**		0.9955	1.0000	1.0000	1.0000	1.0000	1.0000	1.0000	1.0000
**EELM - Bagging**		1.0000	1.0000	1.0000	1.0000	1.0000	1.0000	1.0000	1.0000
**EELM - Stacking**	1.0000	1.0000	1.0000	1.0000	1.0000	1.0000	1.0000	1.0000	1.0000
**EELM - Boosting**		0.9959	0.9996	1.0000	1.0000	1.0000	1.0000	1.0000	1.0000
**EANN - Bagging**		0.9054	0.9248	0.9526	0.9577	0.9645	0.9659	0.9630	0.9584
**EANN - Stacking**	0.7617	0.9271	0.9697	0.9841	0.9907	0.9932	0.9946	0.9976	0.9993
**EANN - Boosting**		0.9856	1.0000	1.0000	1.0000	1.0000	1.0000	1.0000	1.0000

κ (Testing)									
**Model \ Size**	**1**	**5**	**10**	**15**	**20**	**25**	**30**	**40**	**50**
**ELR - Bagging**		0.7636	0.7712	0.7883	0.7846	0.7890	0.7800	0.7876	0.7886
**ELR - Stacking**	0.6914	0.7551	0.7643	0.7811	0.7710	0.7737	0.7638	0.7788	0.7834
**ELR - Boosting**		0.7290	0.7545	0.7609	0.7809	0.7770	0.7777	0.7893	0.7808
**ESVM- Bagging**		0.7449	0.7586	0.7837	0.7696	0.7820	0.7730	0.7805	0.7813
**ESVM - Stacking**	0.6567	0.7406	0.7438	0.7527	0.7470	0.7640	0.7679	0.7495	0.7636
**ESVM - Boosting**		0.7308	0.7490	0.7279	0.7371	0.7594	0.7598	0.7618	0.7629
**RF - Bagging**		0.5144	0.5321	0.5136	0.5510	0.5366	0.5438	0.5512	0.5530
**RF - Stacking**	0.4510	0.5114	0.5367	0.5131	0.5594	0.5617	0.5736	0.5704	0.5660
**RF - Boosting**		0.5598	0.5951	0.6113	0.6145	0.6323	0.6238	0.6344	0.6371
**EELM - Bagging**		0.5502	0.5614	0.5539	0.5571	0.5582	0.5566	0.5524	0.5549
**EELM - Stacking**	0.5324	0.5502	0.5543	0.5539	0.5540	0.5582	0.5535	0.5505	0.5540
**EELM - Boosting**		0.5147	0.5190	0.5150	0.5117	0.5095	0.5151	0.5137	0.5139
**EANN - Bagging**		0.6920	0.7117	0.7593	0.7430	0.7717	0.7669	0.7559	0.7610
**EANN - Stacking**	0.5835	0.6918	0.7291	0.7339	0.7418	0.7215	0.7517	0.7492	0.7455
**EANN - Boosting**		0.6971	0.7607	0.7765	0.7822	0.7816	0.7911	0.7986	0.8103

Supplementary Table S3. Average training (top) and testing (bottom) results of the ensemble models with varying data availability.

κ (Training)								
**Model**	**20%**	**30%**	**40%**	**50%**	**60%**	**70%**	**80%**	**90%**
**ELR - Bagging**	0.9979	1.0000	0.9974	0.9913	0.9757	0.9655	0.9593	0.9511
**ELR - Stacking**	1.0000	1.0000	1.0000	0.9962	0.9934	0.9811	0.9823	0.9770
**ELR - Boosting**	1.0000	1.0000	1.0000	1.0000	1.0000	1.0000	1.0000	1.0000
**ESVM- Bagging**	0.9768	0.9298	0.8831	0.8508	0.8275	0.7973	0.7948	0.7773
**ESVM - Stacking**	1.0000	0.9920	0.9641	0.9405	0.9333	0.9026	0.8878	0.8817
**ESVM - Boosting**	1.0000	1.0000	1.0000	1.0000	1.0000	0.9995	1.0000	0.9940
**RF - Bagging**	0.8797	0.9092	0.9307	0.9374	0.9307	0.9359	0.9363	0.9416
**RF - Stacking**	0.9547	0.9857	0.9919	0.9903	0.9924	0.9928	0.9922	0.9889
**RF - Boosting**	1.0000	1.0000	1.0000	1.0000	1.0000	1.0000	1.0000	1.0000
**EELM - Bagging**	1.0000	1.0000	1.0000	1.0000	1.0000	1.0000	1.0000	1.0000
**EELM - Stacking**	1.0000	1.0000	1.0000	1.0000	1.0000	1.0000	1.0000	1.0000
**EELM - Boosting**	1.0000	1.0000	1.0000	1.0000	1.0000	1.0000	1.0000	1.0000
**EANN - Bagging**	0.9436	0.9226	0.9191	0.9430	0.9379	0.9549	0.9527	0.9558
**EANN - Stacking**	1.0000	0.9988	0.9975	0.9993	0.9989	0.9991	0.9990	0.9985
**EANN - Boosting**	1.0000	1.0000	1.0000	1.0000	1.0000	1.0000	1.0000	1.0000

κ (Testing)								
**Model**	**20%**	**30%**	**40%**	**50%**	**60%**	**70%**	**80%**	**90%**
**ELR - Bagging**	0.3928	0.5735	0.6401	0.7086	0.7248	0.7539	0.7763	0.7825
**ELR - Stacking**	0.4509	0.5530	0.6156	0.6636	0.6903	0.7364	0.7611	0.7696
**ELR - Boosting**	0.4013	0.5419	0.6172	0.6845	0.7047	0.7418	0.7560	0.7738
**ESVM- Bagging**	0.5000	0.5513	0.6351	0.6522	0.7187	0.7083	0.7734	0.7461
**ESVM - Stacking**	0.4968	0.5615	0.6005	0.6580	0.6953	0.7261	0.7372	0.7466
**ESVM - Boosting**	0.4880	0.5789	0.6170	0.6814	0.7179	0.7310	0.7527	0.7601
**RF - Bagging**	0.3700	0.4015	0.4315	0.4841	0.4621	0.5434	0.5391	0.5389
**RF - Stacking**	0.3538	0.4207	0.4665	0.5079	0.4896	0.5412	0.5525	0.5526
**RF - Boosting**	0.4013	0.4668	0.5109	0.5495	0.5867	0.6219	0.6296	0.6452
**EELM - Bagging**	0.3177	0.3510	0.3832	0.4008	0.4340	0.4898	0.5085	0.5305
**EELM - Stacking**	0.3157	0.3495	0.3829	0.4006	0.4339	0.4891	0.5078	0.5259
**EELM - Boosting**	0.3625	0.3621	0.4098	0.4236	0.4507	0.4742	0.4899	0.5038
**EANN - Bagging**	0.4470	0.4988	0.5399	0.5921	0.6505	0.7105	0.7393	0.7553
**EANN - Stacking**	0.4780	0.5310	0.5540	0.6175	0.6645	0.6838	0.7201	0.7222
**EANN - Boosting**	0.4799	0.5653	0.6087	0.6569	0.7001	0.7411	0.7552	0.7684
